# Efficacy and safety of Shengjiang Xiexin decoction in prophylaxis of chemotherapy-related diarrhea in small cell lung cancer patients: study protocol for a multicenter randomized controlled trial

**DOI:** 10.1186/s13063-020-04275-5

**Published:** 2020-05-01

**Authors:** Chao Deng, Yanni Lou, Yu Gao, Bo Deng, Fei Su, Liqun Jia

**Affiliations:** 1 Department of medical oncology, integrated traditional Chinese and Western Medicine, China-Japan Friendship Hospital, No.2, East Street, Ying Hua Yuan, Chao Yang District, Beijing, 100029 China; 2 Beijing University of Chinese Medicine, No.11, East Road, North Third Ring Road, Chao Yang District, Beijing, 100029 China

**Keywords:** Chemotherapy-related diarrhea, Prophylaxis, Shengjiang Xiexin decoction, Randomized controlled trial

## Abstract

**Background:**

Diarrhea is a common adverse reaction in patients with cancer receiving chemotherapy, for which there is currently no effective method of treatment. Shengjiang Xiexin decoction (SXD), a classic traditional Chinese medicine (TCM) formula, has shown efficacy in alleviating irinotecan-induced diarrhea in preliminary clinical studies. The current study is designed to assess the efficacy and safety of SXD for prophylaxis against irinotecan-induced diarrhea. Additionally, we employ a new approach to analyze and evaluate the data based on the patients’ uridine diphosphate glucuronosyltransferase 1A1 (UGT1A1) genotype, which predicts the risk of diarrhea.

**Methods and design:**

A prospective, double-blind, randomized, placebo-controlled trial will be conducted in patients with small cell lung cancer (SCLC) from five hospitals in China. For this study, 100 irinotecan-naïve patients will be randomly allocated to either the SXD or placebo arms in a 1:1 ratio. Stratified randomization will be used to divide subjects by UGT1A1 genotype into groups with differing risk of diarrhea. The trial will consist of two cycles of chemotherapy with 14 days of oral administration of SXD or placebo administered beginning between 3 days before and up to 11 days after initiation of each chemotherapy cycle. The primary study outcome is the incidence of diarrhea. Secondary outcomes include the degree of diarrhea, the degree of neutropenia, the rate of alterations in chemotherapy regimens, the amount of antidiarrheal drug taken, the rate of hospitalization, and evaluation of chemotherapy efficacy.

**Discussion:**

This study is the first to use the UGT1A1 genotype to stratify patients into groups based on their risk of diarrhea, and to provide a complete assessment of chemotherapy-related diarrhea (CRD), including records of diarrhea duration, grading the severity of diarrhea, and evaluating concomitant symptoms. Study results will provide high-level clinical evidence on the use of SXD as prophylaxis for CRD.

**Trial registration:**

Chinese Clinical Trial Register: ChiCTR1800018490. Registered on 20 September 2018. Retrospectively registered. http://www.chictr.org.cn/edit.aspx?pid=25250&htm=4c

## Background

Chemotherapy-related diarrhea (CRD) is a common adverse reaction in oncotherapy [1]. Treatment often requires reducing the chemotherapeutic dose and delaying or interrupting the chemotherapy schedule due to severe and potentially life-threatening dehydration and electrolyte disruption [2]. When that happens, patients often require hospitalization to replenish fluids and electrolytes, which increases patients’ medical expenses. As a consequence, the effects of chemotherapy may be attenuated and the patient’s financial burden may be increased. Irinotecan is a first-line or second-line therapeutic agent for small cell lung cancer (SCLC) that often induces diarrhea. The incidence of all degrees of attendant diarrhea is 50–80% [3]; the incidence of severe diarrhea (grade 3–4) is 11–32 %[4, 5]. In patients with the UGT1A1 gene mutation, the risk of severe diarrhea increases significantly [6].

The mechanism underlying irinotecan-induced diarrhea is not yet clear [7]. It is accepted that the UGT1A1 gene polymorphism test can be used to predict the risk of diarrhea based on the metabolic sequelae of irinotecan in vivo [8]. A study of the correlation between UGT1A1 gene polymorphisms in Chinese patients and irinotecan toxicity showed that UGT1A1*28 genotypes are more involved in the development of diarrhea. Thus, the incidence of severe diarrhea in patients accepting irinotecan with wild type UGT1A1*28, heterozygous UGT1A1*28, and homozygous UGT1A1*28 was 1.28% (1/78), 31.5% (6/19), and 66.7% (4/6), respectively [9]. It is thus clear that the UGT1A1 gene is important in irinotecan-induced diarrhea. However, previous studies rarely included the UGT1A1 genotype as an influencing factor when grouping, which will produce selection bias and inaccurate results.

The currently recommended treatments for CRD, which are based on specialist consensus and guidelines, have important deficiencies, such as dubious efficacy [10]. easy invalidation [11], or accompanying side effects [12, 13], and none have been approved for clinical benefit. Effective prophylaxis is also lacking.

SXD is recorded in the traditional Chinese medicine (TCM) classic “Treatise on Febrile Diseases” from 2000 years ago. It is typically administered for the treatment of inflammatory bowel disease [14]. In our previous study, we found that SXD significantly reduced irinotecan-induced gastrointestinal toxicity, especially in the presence of the UGT1A1 mutation. It is well-known that toxicity with the mutant type is more serious than with the wild type. However, no significant differences in toxicity were noted between the mutant and the wild type when SXD was administered (*P* > 0.05) [15]. In addition, we established a model of diarrhea in the rat to explore the possible mechanisms of the antidiarrheal action of SXD. We found that treatment with SXD was associated with an inhibitory effect on intestinal apoptosis combined with a promotional effect on intestinal cell proliferation, owing to enhanced maintenance of intestinal stem cells (ISCs) [16]. Another animal study showed that SXD altered the pharmacokinetics of irinotecan and its metabolites to alleviate irinotecan-induced diarrhea, specifically by reducing the hepatic transporters multidrug resistant-associated protein-2 (Mrp-2) and P-glycoprotein (P-gp) and by upregulating UGT1A1 in the liver and jejunum [17].

We designed this study because there have been no large-scale clinical trials and little high-level clinical evidence on the treatment and prevention of CRD. The aim of our study is to evaluate the efficacy and safety of SXD prophylaxis against irinotecan-induced diarrhea in patients with SCLC, specifically that involving the UGT1A1 mutation as determined by genetic analysis before initiating chemotherapy. In this way, we hope to balance the groups by reducing the bias otherwise caused by different levels of risk of CRD in different genotypes. Meanwhile, we will also evaluate whether SXD has an impact on the efficacy of chemotherapy.

## Methods and designs

### Study design

The study will be conducted in five hospitals in Beijing, China, including China-Japan Friendship Hospital (Ethical Approval Document No. 2018–82-K57), Beijing Friendship Hospital Affiliated with Capital Medical University (2019-P2–016-02), Beijing Hospital of TCM Affiliated with Capital Medical University (2018BL-057-02), Wangjing Hospital of China Academy of Traditional Chinese Medicine (WJEC-KT-2018-045-P001), and Beijing Chaoyang Hospital Affiliated with Capital Medical University (2018-KE-313).

**Fig. 1:**
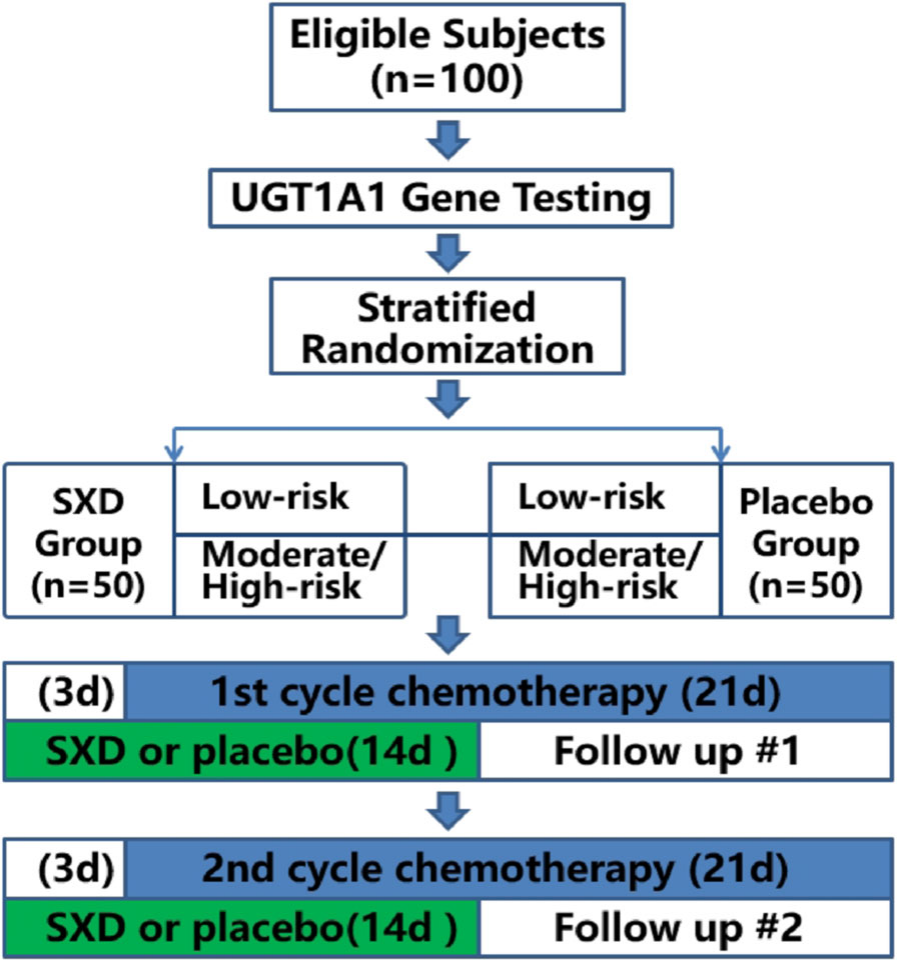
The study design flow chart. SXD, Shengjiang Xiexin decoction; d, day

**Fig. 2:**
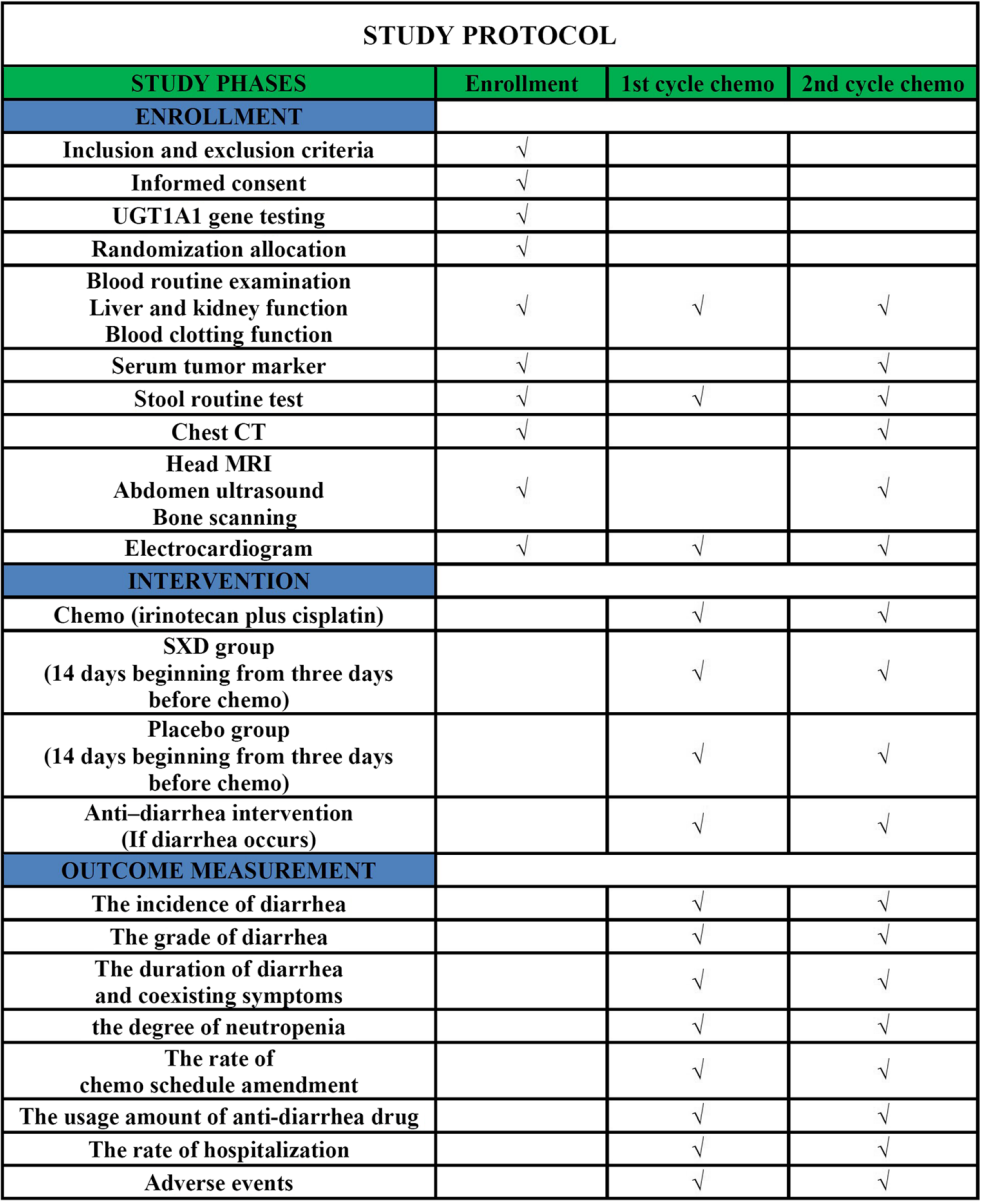
The schedule of study phases; chemo, chemotherapy; CT, computed tomography; MRI, magnetic resonance imaging; SXD, Shengjiang Xiexin decoction

The study design flow chart is shown in Fig. 1. The trial will use stratified random grouping. Eligible subjects will first undergo UGT1A1 gene testing and be stratified to one of two levels, low or moderate-to-high risk of CRD, after which they will be randomly allocated to the SXD treatment cohort or the placebo cohort within their risk group. Patients will be excluded if UGT1A1 testing fails for any reason. The trial will include two cycles of chemotherapy (21 days per cycle), and each cycle will consist of 14 days of oral administration of SXD or placebo beginning 3 days before initiation of chemotherapy, with a follow-up visit from the initiation of chemotherapy. The trial schedule, including patient enrollment, interventions, and outcome measurements is shown in Fig. 2.

### Sample size

The study proposes that SXD can reduce the occurrence of irinotecan-related diarrhea in patients with SCLC, which was the working hypothesis we used to calculate sample size. This study was designed as a superiority trial and sample size calculations are based on the primary outcome measurement - the incidence and percentage of CRD. Based on the results of our preliminary observational study and previous literature [18], the incidence of CRD is 37.5% (6/16) with SXD and 63.4% without SXD, respectively.

In our study, the ratio of subjects will be 1:1 in both the SXD and control groups. Sample size calculations were performed using PASS software, version 15.0, for a superiority test for two independent, randomized samples. With a significance level of 5% (α), a two-sided test, and power of 80% (1 − β), the required sample size was determined to be approximately 40 per group. Considering an estimated dropout rate of 20%, 50 subjects will be required per group, giving a total of 100 subjects.

### Inclusion and exclusion criteria

The inclusion criteria are as follows:

1. Patients with SCLC diagnosed by pathology testing;
2. Irinotecan-naïve subjects who will be undergoing chemotherapy with irinotecan for the first time;
3. No chemotherapy administered in the 3 months before patient enrollment;
4. Age range between 18 and 70 years;
5. Body mass index (BMI) > 20 kg/m^2^ and < 30 kg/m^2^;
6. Patients with a score of 0–2 on the Eastern Cooperative Oncology Group (ECOG) assessment;
7. Estimated patient survival duration ≥ 6 months;
8. Patients with no heart, liver, kidney, or other major organ dysfunction, and the following laboratory values: neutrophils > 1.5 × 10^9^/L; platelets > 100 × 10^9^/L; hemoglobin > 90 g/L; bilirubin within normal limits or < 1.5 × the upper limit of normal (ULN); aspartate aminotransferase (AST)/alanine aminotransferase (ALT) < 2.5 × ULN; serum creatinine < 1.5 × ULN; endogenous creatinine clearance (CCR) ≥ 60 mL/min (calculated using the Cockcroft-Gault formula);
9. Patients able to fully understand the aims and parameters of this study and provide signed, informed consent.

The exclusion criteria are as follows:

1. Patients with diarrhea caused by primary diseases (such as acute and chronic enteritis or inflammatory bowel disease), radioactive enteritis, long-term use of laxatives or drugs resulting in diarrhea (including antibiotics);
2. Pelvic radiotherapy administered in the 2 weeks before study initiation;
3. Intolerance to chemotherapy due to serious organic diseases or infections, such as heart, lung, or kidney failure in a decompensated stage;
4. Patients whose UGT1A1 gene cannot be assessed;
5. Pregnant or lactating women;
6. Patients participating in other clinical trials currently or within the 4 weeks before study initiation;
7. History of diagnosed psychiatric condition that may affect the patient’s ability to understand the study parameters and provide informed consent.

### Recruitment of subjects

Recruitment will mainly be conducted by means of posters and online advertisements. Posters will be displayed in the outpatient clinics and inpatient wards of each center, and the advertisements will be placed on each center’s website. Both the posters and advertisements will include the study profile, inclusion/exclusion criteria, interventions, and contact information. Physicians at each center will identify prospective patients. After their eligibility is confirmed, they will sign informed consent forms before they are enrolled.

### Subject withdrawal criteria

Study participation will be terminated if:

1. A severe adverse event (AE) occurs during chemotherapy;
2. The chemotherapy schedule requires modification because of severe adverse reactions to chemotherapy;
3. Subjects do not comply with this study’s requirements.

### UGT1A1 gene testing

UGT1A1 gene testing will be performed by a single, external, biology laboratory (Beijing Ruibo Xingke Biotechnology Co. Ltd., Beijing, China). At least 2.5 mL of peripheral venous blood will be obtained from each eligible participant using a 4-mL EDTA anticoagulant blood collection tube. The blood sample will be stored and transported at − 20 °C. Genomic DNA will be extracted from each blood sample and UGT1A1*28 genotypes will be amplified by polymerase chain reaction (PCR). Gene polymorphisms will be detected using direct sequencing.

### Randomization and blinding

The relative risk of irinotecan-induced diarrhea for different UGT1A1 gene polymorphisms is wild type (TA6/6) < heterozygous mutant (TA6/7) < homozygous mutant (TA7/7). Based on this, we will perform stratified UGT1A1 genotype randomization. Eligible participants with the UGT1A1 gene will first be stratified into one of two groups - low or moderate-to-high risk of CRD - after which they will be randomized to the SXD cohort or placebo cohort for each level of risk.

Randomization will be implemented by an external agency specializing in the conduct of randomized clinical trials (Beijing Yin Rui Da Medicine Technology Co. Ltd., Beijing, China). The agency will not participate in data management or statistical analysis. Statistical Analysis System (SAS) software, version 9.4, will be utilized to stratify randomization.

The stratified ratio of participants in the low risk (TA6/6) and moderate-to-high risk (TA6/7 and TA7/7) groups will be consistent with the methods used in prior studies. In one previous study, UGT1A1 gene detection in 141 patients with cancer treated with irinotecan showed that the distribution of UGT1A1*28 TA6/6, TA6/7, and TA7/7 genotypes was 74.4% (*n* = 105), 23.4% (*n* = 33), and 2.1% (n = 3), respectively [19]. In a separate study of 2093 Chinese patients from more than 15 hospitals in Shandong, the distribution of the UGT1A1*28 TA6/6, TA6/7 and TA7/7 genotypes was 76.5% (*n* = 1601), 22.1% (*n* = 463) and 1.4% (*n* = 29), respectively [20]. The estimated stratum ratio is therefore determined to be 3:1.

The random sequence will be generated using SAS software with an allocation ratio of 1:1. SXD or placebo will be sealed in an opaque paper box labeled by randomization number and allocated based on that number. The participants will be assigned a randomization number and will receive the corresponding package, containing either SXD or the placebo.

All researchers and participants, excluding the researcher in charge of randomization, will be blinded to the assignment until the trial is completed. Emergency letters will be attached to each labeled box that will be kept by a specially assigned person in each center. If participants have severe AEs, the letters will be opened to reveal the participants’ allocated interventions.

### Intervention

All subjects will receive two cycles of irinotecan-based chemotherapy according to the 2018 National Comprehensive Cancer Network (NCCN) Clinical Practice Guideline for SCLC (version 2.0) as follows: intravenous infusion of irinotecan 65 mg/m^2^ body surface area (BSA) and cisplatin 30 mg/m^2^ on days 1 and 8, administered 3 weeks apart.

**Fig. 3:**
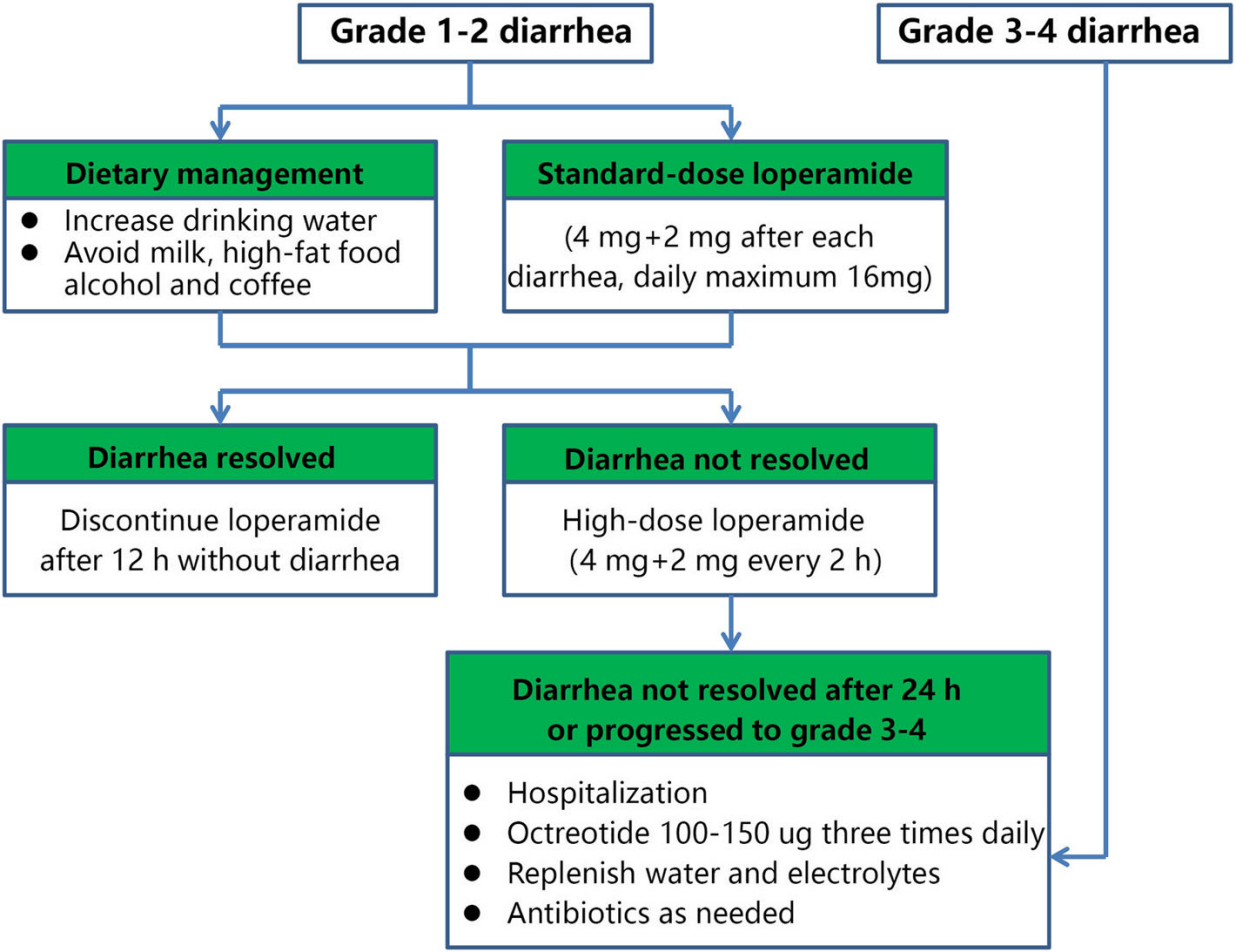
Consensus guideline for the treatment of chemotherapy-related diarrhea

SXD and placebo will be manufactured by the pharmaceutical company New Green Science and Technology Development Co. Ltd., of Sichuan, China, according to Good Manufacturing Practices (GMP) for Pharmaceutical Products, People’s Republic of China. SXD comprises eight Chinese herbs (as shown in Table 1) that will be extracted and concentrated using a standardized Chinese formula. The resulting granules will be packed in aluminum foil bags as is permitted and regulated by the China Food and Drug Administration (CFDA). The placebo will consist of maltodextrin, starch, bitters, and food coloring, and will have a similar appearance, color, weight, taste, smell, and packaging to SXD. The participants will be instructed to dissolve their SXD or placebo and take it as a dose of 3.2 g (per bag) each time, twice daily, beginning 3 days before each cycle of chemotherapy to 11 days after the start of chemotherapy. Participants will be given antidiarrheal medication if they develop diarrhea, according to the CRD specialist consensus and guidelines [5], as detailed in Fig. 3.

**Table 1:** Constituents of Shengjiang Xiexin decoction (SXD)

Chinese name	Botanical latin name	Proportion
Shengjiang	*Rhizoma zingiberis recens*	4
Ganjiang	*Rhizoma zingiberis*	1
Huangqin	*Radix scutellariae*	3
Huanglian	*Rhizoma coptidis*	1
Banxia	*Rhizoma pinelliae*	3
Dangshen	*Radix codonopsis*	3
Dazao	*Fructus jujubae*	4
Gancao	*Radix glycyrrhizae*	3

### Outcome measurement

#### Primary outcome

The primary outcome measure is the incidence and percentage of CRD in each group. CRD is defined as diarrhea occurring > 24 h after irinotecan administration and up to the completion of both chemotherapy cycles.

#### Secondary outcomes

Secondary outcomes include the degree of diarrhea, degree of neutropenia, incidence of chemotherapy regimen alterations, amount and dose of antidiarrheal drugs taken, incidence of hospitalization for diarrhea, and evaluation of chemotherapy efficacy.

Each participant will be supplied with a diary in which they will be instructed to record the time, duration, and coexisting symptoms of diarrhea. The information will then be reported to the researchers. The grade of diarrhea will be assessed for each chemotherapy cycle, and the most serious grade for each participant will be recorded to compare the difference in grades between the SXD and placebo cohorts. The diarrhea grading criteria used will be in compliance with the Common Terminology Criteria for Adverse Events (CTCAE), Version 5.0, released by the National Cancer Institute (NCI), which is detailed in Table 2. In addition, to completely evaluate each episode of diarrhea, participants will be instructed to record in detail the duration of diarrhea and the presence of coexisting symptoms, such as stomachache, abdominal distension, and cramps.

**Table 2:** Diarrhea grading criteria

Grade of diarrhea	Definition
**Grade 1**	Increase of < 4 stools per day from baseline; mild increase in ostomy output compared to baseline
**Grade 2**	Increase of 4–6 stools per day from baseline; moderate increase in ostomy output compared to baseline; limiting instrumental ADL
**Grade 3**	Increase of ≥ 7 stools per day from baseline; hospitalization indicated; severe increase in ostomy output compared to baseline; limited self-care in ADL
**Grade 4**	Life-threatening consequences; urgent intervention indicated
**Grade 5**	Death

*ADL* activities of daily living

The development of diarrhea can affect the chemotherapy dose, duration, and schedule. Therefore, alterations in the chemotherapy schedule will be calculated in each group, including dose reductions, treatment postponement or termination, and other alterations in the preplanned chemotherapy regimen. If serious diarrhea occurs, antidiarrheal drugs and even hospitalization may be required, as determined by the recommended guidelines and expert consensus. The amounts and doses of antidiarrheal drugs prescribed and the incidence of hospitalization will be recorded. Common antidiarrheal drugs are loperamide and berberine hydrochloride [21, 22].

We will also evaluate whether SXD affects the efficacy of chemotherapy. When enrolling in the trial, eligible patients will complete the following baseline examinations: blood tumor markers (neuron-specific enolase (NSE) and pro-gastrin-releasing peptide (Pro-GRP)), chest computed tomography (CT), abdominal ultrasound, head magnetic resonance imaging (MRI), and a bone scan. After two cycles of chemotherapy, these examinations will be performed again and the Response Evaluation Criteria in Solid Tumors (RECIST1.1) will be used to evaluate the result.

Safety outcomes

The safety outcomes include AEs related to chemotherapy and SXD. Hematologic toxicity is a common AE related to the administration of irinotecan plus cisplatin [23]. Therefore, routine blood tests, including quantification of leukocytes, neutrophils, hemoglobin, and blood platelets, will be carried out before and after the day of the chemotherapy infusion (days 1 and 8 in a 21-day cycle). In addition, hepatic and renal function, blood clotting function, and electrocardiograms will be recorded at the same time. If the results are obviously abnormal, or if other severe chemotherapy-related adverse reactions occur, the chemotherapy schedule will be modified as required.

If any serious AEs related to SXD administration occur during the trial, the investigator will immediately provide the necessary diagnosis and treatment, and will report each incident to the study principal investigator within 24 h. All AEs will be surveyed and recorded in detail by an independent clinical research associate (CRA) until the participants recover to a stable condition.

### Quality control

To maintain the quality of the study, the directors at each center will form a steering committee that will review study progress, resolve any problems, and guide the process of implementation. All investigators from each center will receive training to unify study criteria and procedures across centers. Two investigators will collect study data simultaneously. To reduce subjective bias, two clinical investigators will evaluate the diarrhea data independently during the follow-up period. Two chief oncologists will evaluate chemotherapy response, and if necessary they will modify the predetermined chemotherapy regimens. Any discrepancies in their evaluations will be adjudicated by a third chief oncologist.

The CRA in each hospital will manage the data and monitor trial progress according to the protocol by checking the informed consent and case report forms (CRFs) and the original results of the laboratory tests and imaging studies for each participant.

An independent third party (Beijing Qihuang Medicine Clinical Research Center, Beijing, China) will be responsible for monitoring and evaluating the data. They will also audit trial progress every 3 months and report any suggestions for optimizing the protocol and conduct of the study to the steering committee.

### Statistical analysis

The study data will be managed and analyzed by an independent biostatistician using SPSS (Statistic Package for Social Science, v20.0). This study includes four data sets for analysis: intention to treat (ITT), per-protocol set (PPS), full analysis set (FAS), and safety set (SS).

ITT analysis will be used to analyze the primary outcome for SXD efficacy in preventing or reducing the incidence and severity of CRD, and will include all study participants. Any missing primary outcome data will be supplemented by carrying forward the last recorded data.

PPS analysis will be performed to test the robustness of the ITT analysis and will only include participants who strictly follow the protocol and finish the study. The results from the two analyses will be considered credible if they are consistent; otherwise, the results will require sufficient discussion and explanation. Furthermore, is compliance is too low, CACE analysis will be implemented to provide an unbiased and robust estimation of treatment effect. The analysis of secondary outcomes will be based on the results of the PPS analysis.

The FAS analysis will include all randomized participants who completed at least one treatment and one follow up. To analyze the intervention-related AEs, the SS analysis will evaluate all safety outcomes for every randomized participant who received any intervention at least once during the study period.

Continuous data will be presented as the mean and standard deviation (SD), and noncontinuous data (including categorical or ranked data) will be presented as count and number (percentage). We will analyze categorical data as the risk ratio (RR) with 95% confidence intervals (CI), and analyze continuous data as mean differences (MD) and their 95% CI.

The incidence of diarrhea, modification rate of the chemotherapy regimen, and incidence of hospitalization are categorical data. The chi-square test or Fisher’s exact test will be conducted to compare the differences in those data between two groups. The Wilcoxon rank sum test will be conducted to evaluate the difference in ranked data between the two groups, which includes the grade of diarrhea and evaluation of chemotherapy efficacy. Repeated-measures analysis of variance (ANOVA) will be used to compare continuous values such as the use of antidiarrheal drugs. In all tests, a *P* value of < 0.05 will be considered statistically significant.

## Discussion

Diarrhea induced by chemotherapy can affect the design and completion of chemotherapy regimens, lower the quality of life in patients with cancer, and increase medical expenses [24]. Loperamide and octreotide are currently recommended to treat CRD, as recommended by expert consensus. However, the effect of loperamide is limited [11], and there is a risk of cardiac arrhythmia when the dose and frequency are too high [12, 13]. Octreotide is not reliably effective in treating CRD. Additionally, there is currently no recommended means of preventing diarrhea. It is therefore necessary to develop an effective method of preventing or reducing the incidence of CRD that does not have significant adverse side effects. Traditional Chinese herbal medicine has recently gained more attentions for its potential to prevent or treat chemotherapy-induced adverse events [25].

Several classic TCM prescriptions have been shown to have some efficacy in treating or preventing irinotecan-induced diarrhea. For example, Banxia Xiexin decoction was shown to relieve or control diarrhea in patients with recurrent SCLC undergoing irinotecan-based chemotherapy [26]. Another Chinese herbal compound, Huangqin decoction (PHY906), was also found to reduce the incidence of irinotecan-induced diarrhea and decrease the use of loperamide [27]. In one of our previous studies in which oral SXD was administered as adjuvant therapy, the incidence of irinotecan-induced diarrhea in patients with UGT1A1 mutation was found to be nearly equal to that of patients with the wild-type mutation [15].. This effect was more notable among patients with a UGT1A1 mutation who were considered to be at high risk of developing CRD. Therefore, for this study, we included the UGT1A1 gene as a stratified random factor to balance the study groups.

The UGT1A1 polymorphism has a significant relationship with irinotecan-induced toxicity, including both diarrhea and neutropenia [28]. The common main gene mutations are UGT1A1*28 and UGT1A1*6; however, both remain controversial in regard to clinical guidance. A meta-analysis involving 58 studies and 6087 patients has demonstrated the predictive value of UGT1A1*28: patients with TA6/7 and TA7/7 genotypes had a higher incidence of diarrhea and neutropenia than those with the TA6/6 genotype [29]. Other studies found that the UGT1A1*6 polymorphism was associated with a higher risk of severe diarrhea [20, 30]. The differences in the findings of these studies might be due to the discrepancy in irinotecan dose, the type of tumor, and patient age. Another study analyzed both UGT1A1*6 and *28 gene polymorphisms and found that UGT1A1*6 plays a more vital role in hematologic toxicity, whereas UGT1A1*28 is more involved in the development of diarrhea [9]. In addition, compared with western populations, UGT1A1*6 mutations are more common than UGT1A1*28 mutations in Chinese populations [31]. Because UGT1A1*28 polymorphisms have higher specificity, we designed our current study to use UGT1A1*28 as the stratification factor for balancing the groups. Future studies that include both the UGT1A1*28 and UGT1A1*6 polymorphisms may provide a more accurate predictive result.

The time to onset of CRD depends on the drug and schedule. The median time to onset of irinotecan-induced late diarrhea is about 6 days with the 3-week, 350 mg/m^2^ dose. For the weekly schedule and 125 mg/m^2^ dose, the median time to onset is 11 days [4]. Objective tumor response is typically evaluated every two cycles. Thus, we designed this study to encompass two complete chemotherapy cycles.

Previous studies have mainly used the CTCAE diarrhea grading system to evaluate CRD. Because this system only focuses on the frequency of diarrhea, but not duration or concomitant symptoms such as stomachache, abdominal distension, and cramps, it cannot provide a complete assessment of reported incidents of CRD. To address this issue, our study will require each participant to record a detailed diary of all aspects of their diarrhea.

More evidence is needed to determine whether adjuvant therapy with Chinese herbal medicine alters the chemotherapy response. Thus, we have chosen to evaluate chemotherapy efficacy as a secondary outcome in this study.

Finally, we have included testing for the UGT1A1 gene as a means of randomly stratifying groups to balance the groups before they are randomized to treatment or control. This measure could thereby reduce bias in evaluating the efficacy of SXD as prophylaxis for irinotecan-induced diarrhea. The results of this study should help to generate high-level clinical evidence for this Chinese herbal compound as a means of preventing or decreasing the incidence of CRD.

## Supplementary information

**Additional files 1.** Standard protocol items: recommendation for interventional trials (SPIRIT) 2013 checklist: recommended items to address in a clinical trial protocol and related documents.

## Data Availability

Not applicable.

## Abbreviations

ADL: Activities of daily living
AEs: Adverse events
CFDA: China Food and Drug Administration
CRA: Clinical research associate
CRD: Chemotherapy-related diarrhea
CRA: Clinical research associate
CRF: Case report form
CT: Computed tomography
CTCAE: Common Terminology Criteria for Adverse Events
ECOG: Eastern Cooperative Oncology Group
FAS: Full analysis set
GMP: Good Manufacturing Practice
ISCs: Intestinal stem cells
ITT: Intention to treat
MRI: Magnetic resonance imaging
NCCN: National Comprehensive Cancer Network
NCI: National Cancer Institute
PCR: Polymerase chain reaction
PI: Principal investigator
PPS: Per-protocol set
RECIST: Response Evaluation Criteria in Solid Tumors
SCLC: Small cell lung cancer
SPSS: Statistic Package for Social Science
SS: Safety set
SXD: Shengjiang Xiexin decoction
ULN: Upper limit of normal
